# Andrographolide induces anti-SARS-CoV-2 response through host-directed mechanism: an *in silico* study

**DOI:** 10.2217/fvl-2021-0171

**Published:** 2022-07-04

**Authors:** Bhabani Shankar Das, Nabarun Chandra Das, Shasank Sekhar Swain, Suprabhat Mukherjee, Debapriya Bhattacharya

**Affiliations:** ^1^Centre for Biotechnology, School of Pharmaceutical Sciences, Siksha ‘O’ Anusandhan (Deemed to be University), Bhubaneswar, 751003, Odisha, India.; ^2^Integrative Biochemistry & Immunology Laboratory, Department of Animal Science, Kazi Nazrul University, Asansol, 713340, West Bengal, India.; ^3^Division of Microbiology & NCDs, ICMR-Regional Medical Research Centre, Bhubaneswar, 751023, Odisha, India.

**Keywords:** andrographolide, host directed therapy, immunoregulator, molecular modeling, SARS-CoV-2, viral entry receptors

## Abstract

**Aim:** Considering the present alarming situation of COVID-19 pandemic, we concentrated on evaluating the efficacy of a novel natural antiviral drug-candidate andrographolide against SARS-CoV-2 through an *in silico* model of study. **Materials & methods:** Interaction of andrographolide against the major host molecules that are responsible for SARS-CoV-2 pathogenesis were determined using bio-computational tools, in other words, molecular docking, molecular dynamics simulation and pharmacodynamics–pharmacokinetics analysis. **Result:** Computational findings represent that andrographolide efficiently interacts with the major human–host-associated putative drug-targets of viral-entry points like furin (-10.54 kcal/mol), TMPRSS-2 (-9.50 kcal/mol), ACE2 (-8.99 kcal/mol) and Cathepsin L (-8.98 kcal/mol). Moreover, it also blocks the inflammatory regulators including TLR4-MD2 and IL-6, which promote virus-induced inflammation leading to cytokine storm in the host body. **Conclusion:** This work elucidates that, the candidature of andrographolide can be utilized as a potent natural agent for the therapeutic intervention of SARS-CoV-2 through host-directed treatment.

The COVID-19 pandemic has become a superior alarming situation in this present day-to-day world. In particular, the second and third waves of SARS-CoV-2 infection have been found spreading across the different countries around the globe. Although, this viral outbreak was first detected in Wuhan, China in the last week of December 2019, the WHO formally declared the COVID-19 a public health emergency of international concern (PHEIC) on 30 January 2020, and officially termed it as SARS-CoV-2. Till 4 April 2022, a total of 486 million confirmed COVID-19 infected and 6 million death cases have been accounted for in this pandemic [1]. In India, more than 43 million people were infected and 0.5 million individuals died from this pandemic viral disease as of 4 April 2022 [1]. Notably, after dropping in infection rate from September 2020 to March 2021, more than 0.3 million people are being infected in India and indicating the arrival of new strains of SARS-CoV-2 [2]. Clinically, the major pathogenic hallmark of SARS-CoV-2 contagion is a severe acute respiratory syndrome that involves acute pneumonia and ‘cytokine storm’ following multi-organ failure in the infected individuals [3]. Additionally, it has been reported to be more communicable than other viruses of the Coronaviridae family [4–7]. Spike glycoprotein (S), membrane protein (M), nucleocapsid protein (N), envelope protein (E) and non-structural proteins such as 3CLpro, PLpro, RdRp and helicase are the key organizational proteins of SARS-CoV-2 and considered to be the promising targets for developing anti-COVID drugs [8]. Out of the various chemotherapeutic approaches, host-directed therapy has emerged as an alternative approach for controlling the SARS-CoV-2 infection and subsequent immunopathogenic episodes by targeting the host molecules implicated in SARS-CoV-2 infection and/or used by SARS-CoV-2 for its entry and pathogenesis. In this context, the receptor-binding domain (RBD) of S1 subunit of the viral S protein binds to ACE2 located on human lung cell [9]. After that, priming of S protein is initiated by the host protease enzymes, which cleave S1/S2 to S1 subunit followed by fusion of viral and cellular membrane through S2 subunit [9,10]. Hence, the most straightforward and easiest approach for combating SARS-CoV-2 would be to prevent the virus from entering host cells by targeting the ACE2 (an aminopeptidase) protein, as it is less likely to mutate [11]. The ACE2 receptor has been identified as the key protein for the cellular entry of viral RNA but some other cell-surface proteins also play pivotal roles. Two important host serine proteases, namely, transmembrane serine protease 2 (TMPRSS2) and Cathepsin B and L dissociate ACE2 receptor-bound N-terminal S1 subunit and membrane-fusion at the C-terminal of S2 subunit by cleaving at S1/S2 cleavage site [12,13]. Eventually, Hoffmann *et al.* showed that TMPRSS2 is more essential for S protein priming and SARS-CoV-2 entry [10]. Similarly, Ou *et al.* also reported that other than TMPRSS2, Cathepsin L but not Cathepsin B has a role in priming of S protein in SARS-CoV-2 [14]. Next, cleavage is needed when SARS-CoV-2 virus particles are endocytosed by the host and move into the phagolysosome wherein lysosomal protease furin mediates the cleavage of S2 subunit (S2′ cleavage site) and releases hydrophobic fusion peptide to fuse with host cell membrane [15]. The proprotein convertase (furin) is a type I transmembrane protein expressed near the trans-Golgi network and activated by acidic pH. Furin cleaves the precursors of a broad range of proteins of the preferred consensus sequence ‘Arg-X-X-Arg ↓’ (R-X-X-R, X: any amino acid, ↓: cleavage site) [16]. Moreover, Wu *et al.* have shown that SARS-CoV-2 has repetitive furin cleavage sites in the S protein which probably increases the chances of fusion of virus particles in the host membrane and simultaneously increases infectivity of SARS-CoV-2 [15]. Intriguingly, the pathophysiology of SARS-CoV-2 results from high inflammatory responses due to the increment of several proinflammatory cytokines like TNF-α, G-CSF, IP-10, MCP-1 and MIP-1A in serum of COVID patients [17], and these cytokines result in cytokine storms [18,19]. Moreover, another cytokine IL-6 has been found to be elevated at maximum limit found in non-survivors than survivors [20]. Besides, IL-6 knockout mice displayed minor lung tissue damage upon experimental infection [21]. Clinical studies have suggested that there is an elevation in the neutrophil infiltration in lung alveoli, and in systemic conditions, with total increment of IL-6 and CRP together with lymphopenia occur in COVID-19 patients [22]. Overall, it indicates that SARS-CoV-2 infection activates a substantial amount of innate immune response, which may be due to upregulation of the expression of the proinflammatory cytokines. The expression of proinflammatory cytokines including IL-6 is dependent on the activation of Toll-like receptors (TLRs). Supporting the above concept, previous studies revealed the interaction of S protein with TLR1, TLR4 and TLR6 has been documented as the prime receptor for the S protein to elicit cytokine response [23–25]. Interestingly, major cytokines like IL-6 and TNF-α involved in the severe COVID-19 cases are the downstream products of the TLR4 signaling pathway [26]. However, other TLRs were also found to recognize single-stranded RNA like TLR7, but TLR4 selectively recognizes the viral S protein [25]. Surprisingly, TLR4 also possesses pathogenic role in COVID-19 infection as it signals the activation of inflammasome, expression of the proinflammatory cytokines like IL6, TNF-α, IL1b and NETosis [26,27]. Therefore, blocking of the host molecules for inhibiting viral binding from host cell fusion to cell membrane or suppressing inflammasome formation by targeting the pathologic functions of TLRs or blocking the most pathologic cytokine IL-6 could cease the inflammatory pathogenesis of SARS-CoV-2.

The available therapeutics have been used on a repurposing basis. For example, lopinavir for HIV, hydroxychloroquine for malaria, remdesivir for rheumatism, ivermectin for filariasis, etc. [28,29]. Most of the repurposed drugs used somehow work on the host part to block SARS-CoV-2 infection or inflammation induced by the virus. Therefore, host-directed therapy for SARS-CoV-2 is working to block viral load as well as associated inflammatory pathophysiology. However, most antiviral drugs exhibit adverse side effects on humans [30]. The development of plant-based medicines or treatment strategies with minimal side effects is expected to overcome this shortfall [31]. Natural phytochemicals have made immense contributions to drug discovery against various human diseases including viral diseases [32]. Currently, screening of effective phytochemicals/natural regimens against SARS-CoV-2 is considered to be a major area of focus. To expedite the search for anti-COVID drugs, several computational studies have been explored through advanced computational programming (homology modeling and molecular docking followed by molecular dynamics simulation) to identify potential inhibitors using existing natural compounds against SARS-CoV-2 [33–36]. However, a few of them have focused on identifying potential inhibitors of host factors that could ultimately inhibit the viral infection [36,37].

The present study examines the *in silico* therapeutic efficacy of andrographolide, a compound found in the Indian medicinal plant *Andrographis* *paniculata* (Burm. f.) Nees reported to have potent antiviral properties [38]. Andrographolide is a diterpene molecule of the isoprenoid family and is presently under consideration to become an anti-SARS-CoV-2 molecule with explicit efficiency of blocking the progression of SARS-CoV-2 infection [39] and immunomodulatory effects on host [40]. However, molecular targets and efficacy of the said phytocompound at the molecular level have not been explored to date. Herein, the biophysical interactions and binding efficiency of andrographolide with different host receptors, in other words, ACE2, human proteases TMPRSS2, Cathepsin L and furin along with two innate inflammatory response receptors such as TLR4-MD2 and IL-6 were studied using advanced *in silico* approaches. Alongside these, experimentally proved NF-κB and viral Mpros were also included to determine the efficacy of the compound of our interest [41,42]. In addition, US FDA-approved ivermectin B1b drug was used as a positive control for comparing of the binding activity of andrographolide against SARS-CoV-2 [28]. As a whole, binding efficacy through molecular docking and intermolecular stability of protein–ligand complex by molecular dynamics simulation with pharmacokinetics demonstrate that andrographolide might be used as a drug molecule to restrict the severity of SARS-CoV-2 infection.

## Materials & methods

### Receptor & ligand structure preparation

3D crystal structures of the selected target proteins namely, ACE2 (PDB ID:6M18) [43], Cathepsin L (PDB ID:5MQY) [44], human furin (PDB ID:6HZD) [45], toll-like receptor 4 (TLR4-MD2) (PDB ID:3FXI) [46], IL-6 protein (PDB ID:4O9H) [47], NF-κB (PDB ID:1lKN) [48] and Mpro (PDB ID:7BRO) [49] retrieved from RCSB Protein Data Bank in PDB format (http://www.rcsb.org) [50]. Due to the unavailability of transmembrane serine protease 2 (TMPRSS2) protein structure in the PDB database, a theoretical model of TMPRSS2 from its amino acid sequence (NCBI reference sequence ID: NP_001128571.1) was prepared by homology modeling using MODELLER 9 [51] validated by Ramachandran plot using SAVESv0.6 server (https://saves.mbi.ucla.edu/). Before docking, all the water molecules, non-standard residues, and indigenous ligands were removed from each protein target using Biovia Discovery Studio Visualizer client 2016 software (DSV). AutoDock 4.2 MG tools were used to add polar hydrogen atoms and Kollman charges to each receptor molecule and finally saved in PDB file formats for docking study [52,53].

The phytochemical of our interest, andrographolide was selected, and the structure was retrieved from PubChem (PubChem CID: 5318517) (https://pubchem.ncbi.nlm.nih.gov/). In addition, ivermectin B1b (PubChem CID: 6321425), a commercially used FDA-approved drug was used for the comparative study. The retrieved SDF format of ligand structures were converted to protein data bank file (PDB) format using the DSV software tool [53]. Afterward, the 3D structure of both ligands was further optimized through the AutoDock compilation tool for further use in docking studies [52].

### Molecular docking study

A series of molecular docking experiments were conducted using AutoDock 4.2 software to determine the binding efficiency through molecular interactions of andrographolide with the selected protein targets [52,54]. The structure of ligand (andrographolide) and target proteins prepared in PDB format was translated to PDBQT format using AutoDock 4.2 software. After that, a unique grid box was created to find out the binding pockets of particular amino acids for each targeted protein to get a reliable docking result. Out of ten docking poses, we have selected the best docking pose based on their lowest binding energy (kcal/mol) with the least root mean square deviation (RMSD). Afterward, output files containing 3D structures of protein–ligand interactions at active macromolecule sites were visualized using PyMol visualization software [55]. Furthermore, the effects of the molecular interactions within the protein–ligand complexes, including hydrophobic bonds, hydrogen bonds and their bond lengths of each docking pose, were also analyzed and portrayed in the form of 2D graphical representation using Discovery Studio Visualizer software [54].

### Normal mode analysis study

The normal mode analysis (NMA) is an *in silico* simulation approach that predicts the biophysical stability and conformational flexibility of a protein in a protein–ligand complex. iMods (http://imods.chaconlab.org/), a popular web tool for NMA study delivers several prediction reports upon uploading PDB file of interest [56]. The server predicts and represents effective motions by a vector field, affine-model arrows and modal animation. Moreover, the server also provides plots of B factors to identify flexibility and deformability to represent non-rigidity. In addition, eigenvalue analysis helps to predict the stability of the structure. Altogether server also predicts covariance and connecting matrix to represent variation in molecular movement.

### Molecular dynamics simulation study

To further understand cohesion, intermolecular forces, and energy gap of the topmost protein (furin) with its intended ligand (andrographolide), a molecular dynamics simulation was performed in GROMACS-5.1.5 platform for 100 ns using GROMOS force field [57]. A topology file of the highest protein inhibitors was generated and a model was prepared using Automated Topology Builder (ATB) software, version 3.0 [58]. After that, the protein–ligand complex was positioned in the center of a cubic periodic box and then solvated by adding simple point charge (SPC) water. The system was electrostatically neutralized by adding counter-ions (Na^+^) and solvates (Cl) ions within the protein–ligand complex. The entire system was optimized for energy efficiency by using the steepest algorithm. Furthermore, temperature range of whole device was increased up to 310 K for 50,000 steps with a period of 2-fs steps. Alongside, pressure was calibrated as 1 bar with constant particle number, pressure and temperature. Another parameter, in other words, NVT was also calibrated for simulation of 100 ps for each residue maintaining steady volume and temperature [59]. After that position of both protein and ligand were restrained. The location restriction of protein and ligand was removed and run for a period of 100 ns with 2-fs time steps. The structural coordinates were saved every 2 ps during MD simulation. The pressure was sustained at 1 bar using Parrinello–Rahman pressure coupling process [60]. Temperature and pressure coupling time was kept constant for 2 ps. Furthermore, short-range electrostatic interactions were measured for atom pairs with a cut-off of 1.4 nm, and long-range electrostatic interactions were calculated using a smooth particle-mesh Ewald (sPME) process with fourth-order cubic interpolation and 0.16 nm grid spacing [61]. The LINCS strategy was used to keep restricted all the bonds [62]. Finally, the RMSD, root mean square fluctuation (RMSF), the number of hydrogen bonds, the radius of gyration (Rg) and solvent accessible surface area (SASA) data were transferred to an excel sheet and plotted for further study.

### Prediction of drug-likeness properties & ADMET analysis

The physicochemical properties followed by standardized Lipinski's rule and the ADMET profiles, including water solubility, oral bioavailability, transportation of blood–brain barrier, binding of plasma proteins, carcinogenicity, hepatotoxicity and acute oral toxicity were determined *in silico* using Swiss-ADME (http://www.swissadme.ch/), admetSAR (http://lmmd.ecust.edu.cn/admetsar2) and pkCSM (http://biosig.unimelb.edu.au/pkcsm/prediction) webserver [63,64]. In addition, drug-likeness and pharmacokinetic properties of andrographolide were also studied.

## Results

The present work explores the anti-SARS-CoV-2 efficacy of the natural antiviral compound andrographolide, a bioactive secondary metabolite of *Andrographis paniculata* by targeting certain human proteins associated with viral entry and pathogenesis. Molecular docking and molecular dynamics simulation were used to determine the efficacy of andrographolide on host proteins having critical roles in the infection and immunopathogenesis of SARS-CoV-2. Notably, two known interacting partners of andrographolide human NF-κB and viral Mpro were also reanalyzed in this *in silico* study for comparing the binding affinity of andrographolide. Similarly, the efficacy of andrographolide was also compared with ivermectin B1b, a known FDA-approved drug for treating SARS-CoV-2. Our findings from the present *in silico* study have been discussed in the subsequent sections.

### Interaction of andrographolide with host-related proteins of SARS-CoV-2 pathogenesis

**Figure 1.**
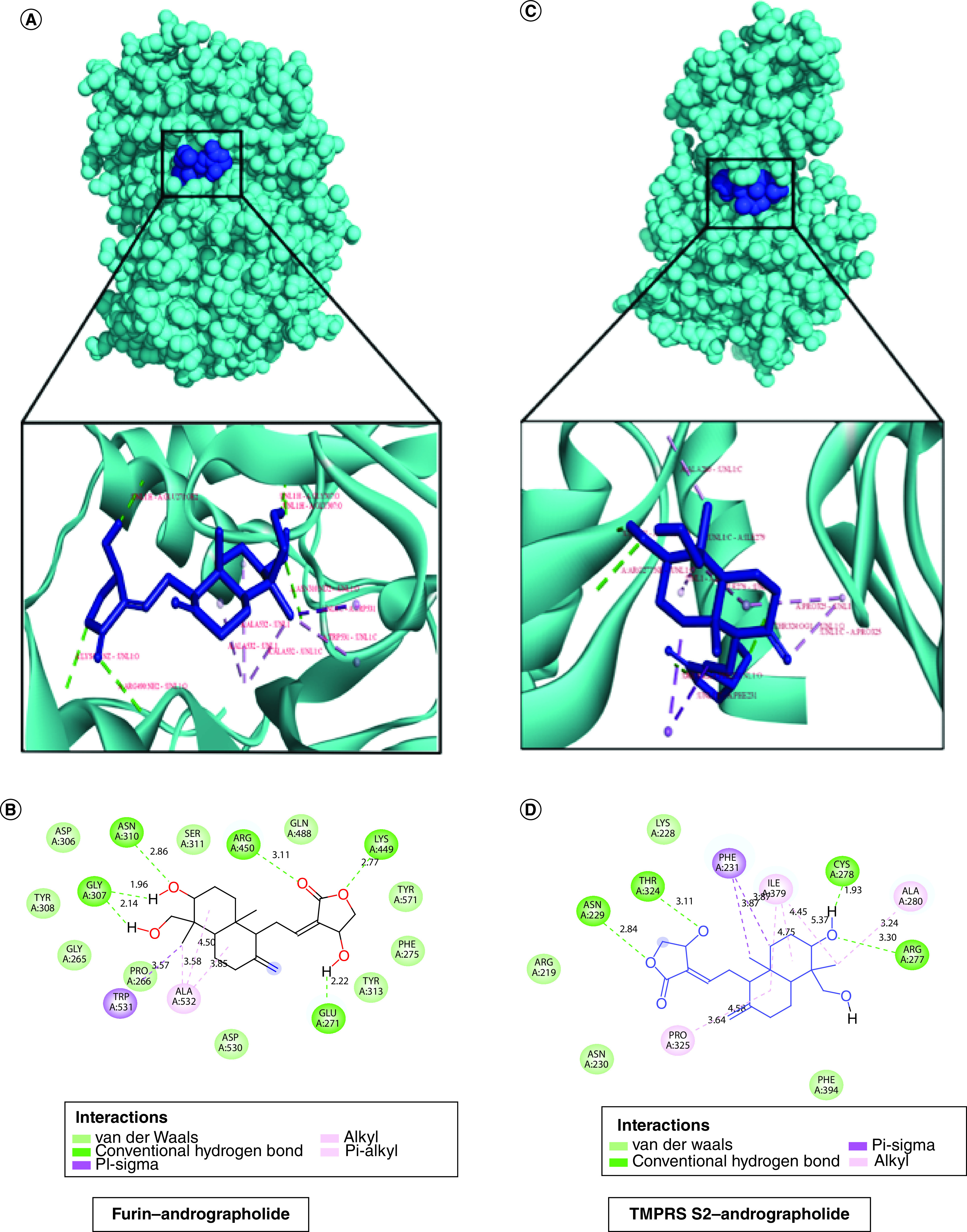
A graphical representation of molecular docking between host molecule furin and TMPRSS2 with phytomolecule andrographolide. **(A)** 3D-docking conformation with their binding modes in between Furin–andrographolide complex protein. **(B)** 2D interaction view of furin–andrographolide complex in Discovery Studio Visualizer. **(C)** 3D-docking view of TMPRSS2–andrographolide complex protein. **(D)** 2D illustration of TMPRSS2–andrographolide complex protein by Discovery Studio Visualizer.

**Figure 2.**
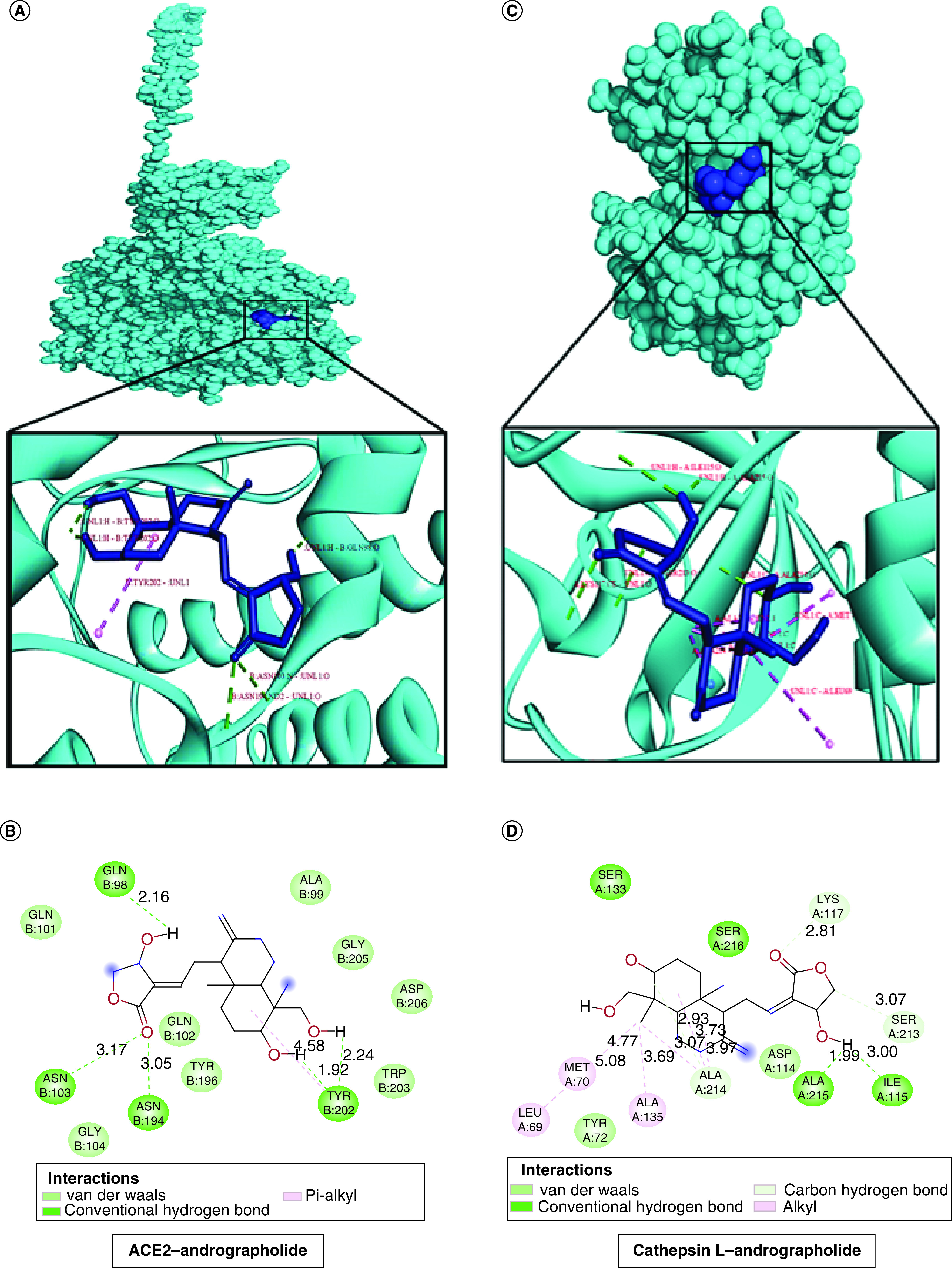
Binding interaction of ACE2 and a host protease Cathepsin L with andrographolide. **(A)** 3D-docking conformation with their binding interactions in between ACE2 and andrographolide. **(B)** 2D view of ACE2–andrographolide complex protein in discovery studio tool. **(C)** 3D-docking view of Cathepsin L with andrographolide protein molecule. **(D)** 2D analyses of Cathepsin L–andrographolide complex protein with their coherent binding modes through Discovery Studio Visualizer.

The molecular docking score of andrographolide against each target protein is documented in Table 1. It was found that the furin enzyme exhibits the most potential interaction with andrographolide molecule (-10.54 kcal/mol) and the interaction is governed by H-bonds (GLY 307, ASN 310, ARG 490, GLU 271 and LYS 449), pi-sigma bond (TRP 531) and pi-alkyl bond (ALA 532) as shown in Figure 1A & B. Afterward, we looked forward to studying the interaction between andrographolide and TMPRSS2 protease enzyme. The docking score of -9.50 kcal/mol was recorded for the TMPRSS2–andrographolide complex that possesses interactive H-bonds (ASN229, THR324, CYS278, ARG277), alkyl bonds (ALA280, ILE279, PRO325) and one pi sigma interactive bond (PHE231) (Figure 1C & D). Subsequently, the compound was found to exhibit hydrophobic interactions with ACE2 receptor protein through GLN98, ASN103, ASN194 and TRY202 residues with a binding affinity value of -8.99 kcal/mol, as shown in Table 1, Figure 2A & B. Additionally, andrographolide was also found to interact with Cathepsin L protein and the binding was found to be mediated by hydrogen bonds (ALA215 and ILE115), carbon–hydrogen bonds (LYS117, SER213 and ALA214), and alkyl bonds (LEU69, MET70, ALA135 and ALA214) with an effective binding energy value of -8.98 kcal/mol as illustrated in Table 1, Figure 2C & D. Therefore, the protein–ligand interaction studies suggest that andrographolide can impair host–virus interactions. Besides these, the binding efficacy was compared with ivermectin B1b taken as a reference drug (represented in Table 1). On the contrary, the andrographolide molecule conveyed a known binding molecule, i.e., NF-κB and viral Mpro, it displayed significant and comparable binding values as depicted in the previous work (Supplementary Table 1).

**Table 1.** Molecular docking analysis of andrographolide and ivermectin B1b against key host associated proteins involved in SARS-COV-2 pathogenesis.

Scientific name	Natural product found in India West Bengal/Odisha name	Compound name	Receptor	Types of bonds	Bonds formed (N)	Amino acid residues	Bond length (Å)	Free energy of binding affinity (ΔG) (kcal/mol)	Inhibition constant (Ki) in nM
***Andrographis paniculata* (Burm. f.) Nees**	Kalmegh/Bhui Nimba	Andrographolide	Furin	Hydrogen	5	ASN307	2.14, 1.96	-10.54	18.70 nM
						ASN310	2.86		
						ARG490	3.11		
						LYS449	2.77		
						GLU271	2.22		
				Pi-Sigma	1	TRP531	3.57		
				Pi-Alkyl	1	ALA532	3.58,4.50, 3.85		
			TMPRSS2	Hydrogen	4	ASN229	2.84	-9.50	109.14 nM
						THR324	3.11		
						CYS278	1.93		
						ARG277	3.30		
				Pi-Sigma	1	PHE231	3.87		
				Alkyl	3	PRO325	3.64		
						ILE279	4.75, 4.45,		
						ALA280	5.37, 3.24, 3.30		
			ACE2	Hydrogen	4	GLN98	2.16	-8.98	261.26 nM
						ASN103	3.17		
				Pi-Alkyl	1	ASN194	3.05		
						TYR202	1.92, 2.24		
						TYR202	4.58		
			Cathepsin L	Hydrogen	2	ALA215	1.99	-8.96	269.20 nM
						ILE115	3.00		
				Alkyl	3	LEU69	5.08		
						MET70	4.77		
						ALA135	3.69		
				Carbon Hydrogen bond	2	LYS117	2.81		
						SER213	3.07		
			TLR4-MD2	Pi-Alkyl	3	PHE76	4.93	-9.88	56.81 nM
						LEU61	5.27, 4.15		
						PHE147	5.45, 3.74		
				Alkyl	4	PHE151	5.42		
						ILE46	4.43, 4.74		
						ILE63	4.15, 5.03		
						ILE44	5.37, 3.58		
			IL6	Hydrogen	2	LUE101	2.11	-8.99	256.45 nM
						GLU42	1.93		
				Alkyl	3	LUE167	5.39		
						LEU39	3.66, 5.12, 3.68		
						ALA112	5.36, 3.01, 4.31		
						ILE224	2.08925		
						GLN241	1.89841		
						ILE224	2.04917		
						ILE224	2.02844		
						GLU222	2.20363		
						GLU225	3.1368		
						GLU49	3.08678		
**5-O-dimethyl-22,23-dihydroavermectin B1b**		Ivermectin B1b	Furin	Hydrogen	2	GLU271	3.16	-14.51	0.02293 nM
						GLU271	3.42		
			TMPRSS2	Hydrogen	4	LYS399	1.69	+5.41	0
						ASN284	2.97		
						LEU285	3.22		
						PHE231	2.80		
				Pi-Lone Pair	1	PHE231	2.41		
				Alkyl	3	ILE279	5.10		
						PRO325	3.62		
						PRO400	3.56		
				Pi-Alkyl	1	PHE394	5.35		
			ACE2	Hydrogen	6	ALA99	2.46	-12.72	0.47498 nM
						LYS562	1.85		
						LYS562	2.36		
						LEU95	2.09		
						ASN194	2.62		
						GLY205	2.98		
				Pi-Alkyl	1	TYR196	4.63		
			Cathepsin L	Hydrogen	4	MET161	2.06	-8.40	701.20 nM
						GLY164	2.80		
						CYS25	2.94		
						GLU159	2.75		
				Alkyl	2	LEU69	4.04		
						ALA214	5.14		
			TLR4-MD2	Hydrogen	1	CYS133	2.94	-14.51	0.02293 nM
				Pi-Sigma	1	TYR102	3.16		
				Pi-Alkyl	1	PHE151	5.14		
				Alkyl	2	CYS133	4.97		
						ILE153	3.43		
			IL6	Hydrogen	4	SER107	1.62	+2.41	0
						SER108	2.60		
						LEU39	1.86		
						GLN102	3.20		
				Alkyl	1	LYS46	5.11		

### Interaction of andrographolide with the host proteins associated with COVID-19 immunopathogenesis

**Figure 3.**
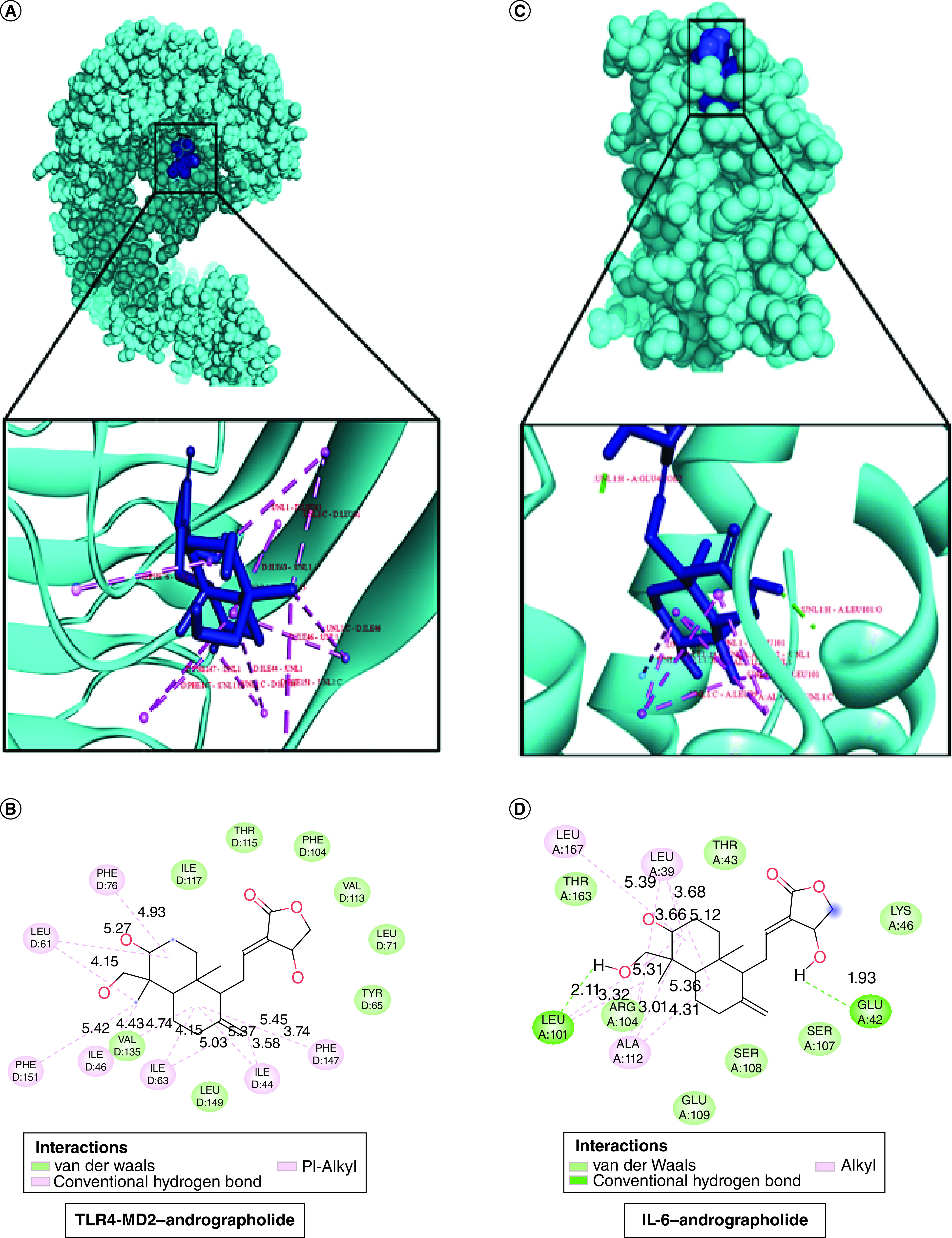
A possible drug-target interaction view of TLR4-MD2 and IL-6 with andrographolide targeted molecule. **(A)** 3D-binding view of TLR4-MD2 with andrographolide molecule. **(B)** 2D-interaction view of TLR4–MD2–andrographolide complex protein. **(C)** 3D-interaction illustration of IL6 with andrographolide targeted molecule. **(D)** 2D-binding view of IL-6- andrographolide complex determined by Discovery Studio Visualizer.

Human ACE2 receptor is vulnerable to SARS-CoV-2 S protein, however, it also utilizes other receptors such as toll-like receptors (TLR), C-lectin type receptors (CLR), and neuropilin-1 (NRP1) to trigger the proliferation and production of pro-inflammatory cytokines, i.e., IL-6 and IL-8, as well as type I and III interferons, to drive immunopathogenic consequences. Therefore, it is necessary to explore the actual binding location of andrographolide molecule with TLR4-MD2 and the pro-inflammatory cytokine IL-6. Surprisingly, our findings indicate that andrographolide molecules possess a strong affinity toward TLR4-MD2 and IL-6 with a strong binding affinity value of -9.88 kcal/mol and -8.96 kcal/mol respectively (Table 1). Furthermore, it was found that the complexes formed through linkages between immunopathogenic proteins and andrographolide which is more efficacious to develop stable interaction. The binding interactions reveal that electrostatic interactions formed by andrographolide with the different residues of TLR4 receptor protein, in other words, PHE76, LEU61, PHE151, PHE147, ILE46, ILE63 and ILE44 play a key role in forming the stable ligand–protein complex. On the other hand, andrographolide was found to form two hydrogen bond forms with IL-6 protein at LEU101, GLU42 residues along with three alkyl bonds at LEU167, LEU39 and ALA112 amino acids (Figure 3A–D). As a consequence, the above mentioned findings conclude that andrographolide could be considered a potential therapeutic drug candidate for inhibition of TLR4 and IL-6 to block SARS-CoV-2-induced immunopathogenicity.

### NMA analyses

**Figure 4.**
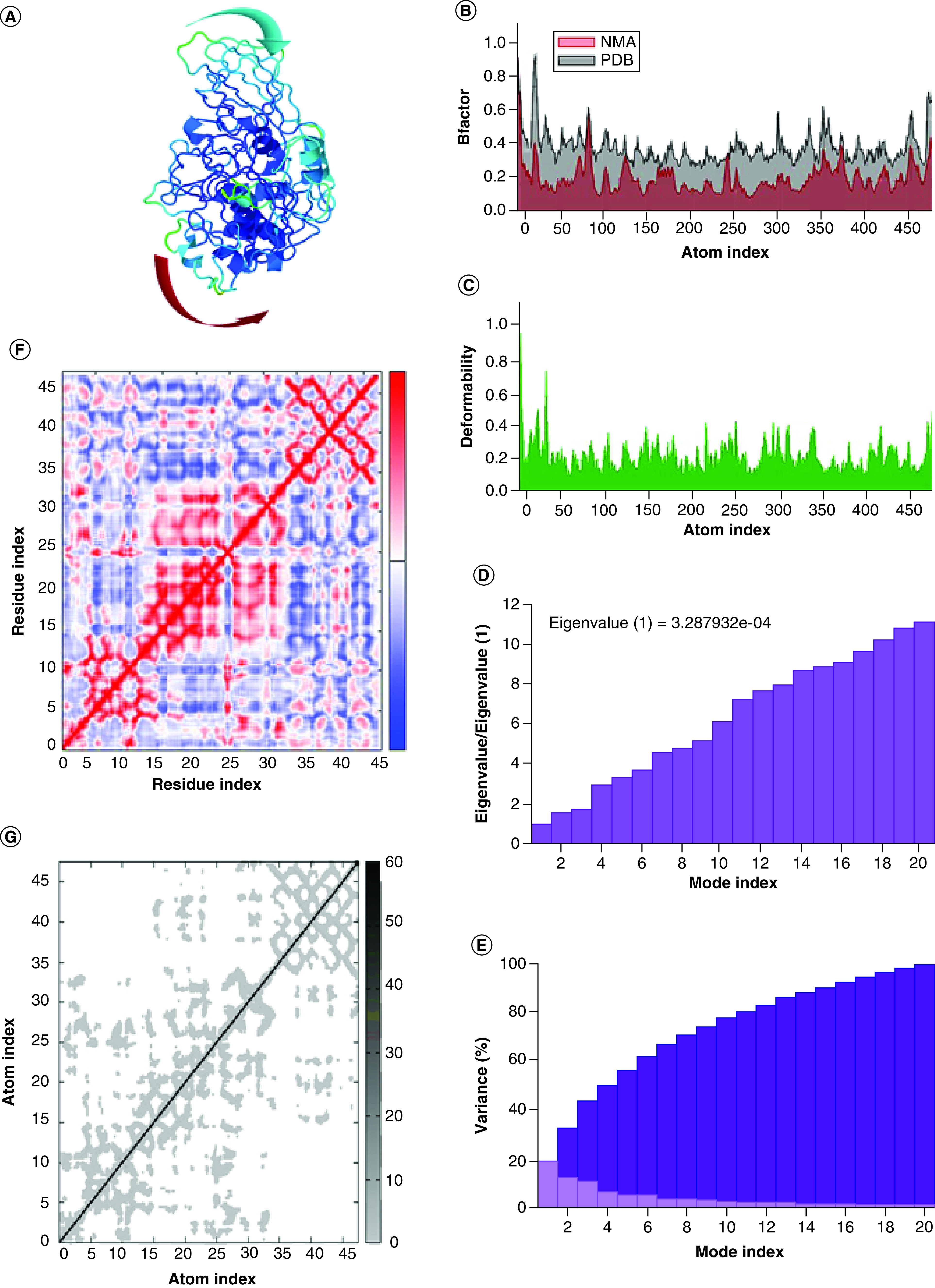
Structural flexibility analyses between human protease furin and phytomolecule andrographolide complex. **(A)** NMA mobility. **(B)** B-factor. **(C)** Deformability. **(D)** Eigenvalues. **(E)** Variance % (red colour remarks individual variances and green colour indicates cumulative variances). **(F)** Co-variance map, correlated (red), uncorrelated (white) or anti- correlated (blue). **(G)** Elastic network (darker gray regions indicate stiffer regions) of the complex.

**Figure 5.**
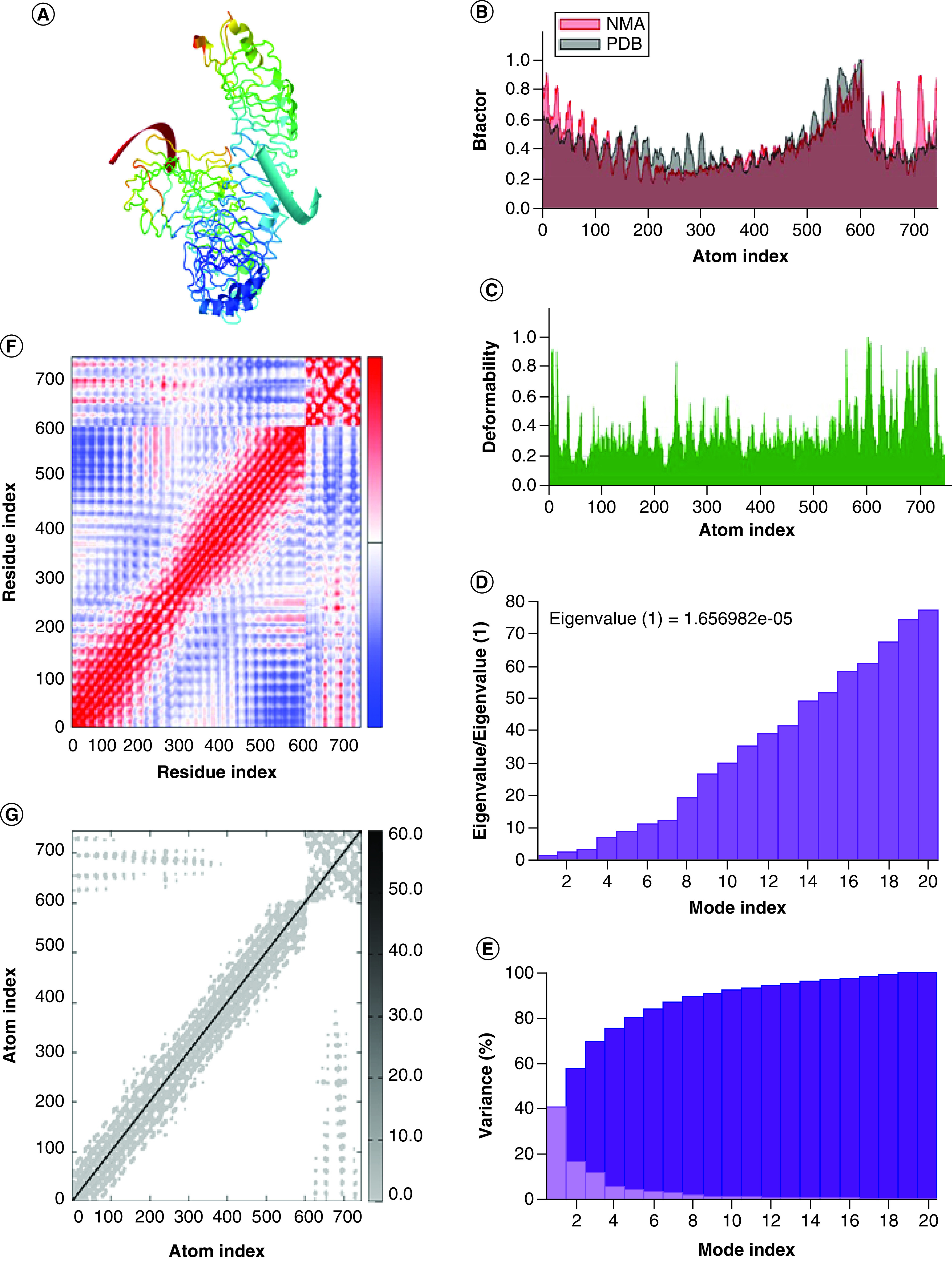
A statistical structural flexibility simulation analysis of TLR4–MD2–andrographolide complex with outcomes of structural flexibility simulation analyses by normal mode analysis between andrographolide and host protein TLR4-MD2 against COVID-19 immunopathogenesis. **(A)** Normal mode analysis mobility. **(B)** B-factor. **(C)** Deformability. **(D)** Eigenvalues. **(E)** Variance % (red colour remarks individual variances and green colour indicates cumulative variances). **(F)** Covariance map, correlated (red), uncorrelated (white) or anti- correlated (blue). **(G)** Elastic network (darker gray regions indicate stiffer regions) of protein complex.

**Figure 6.**
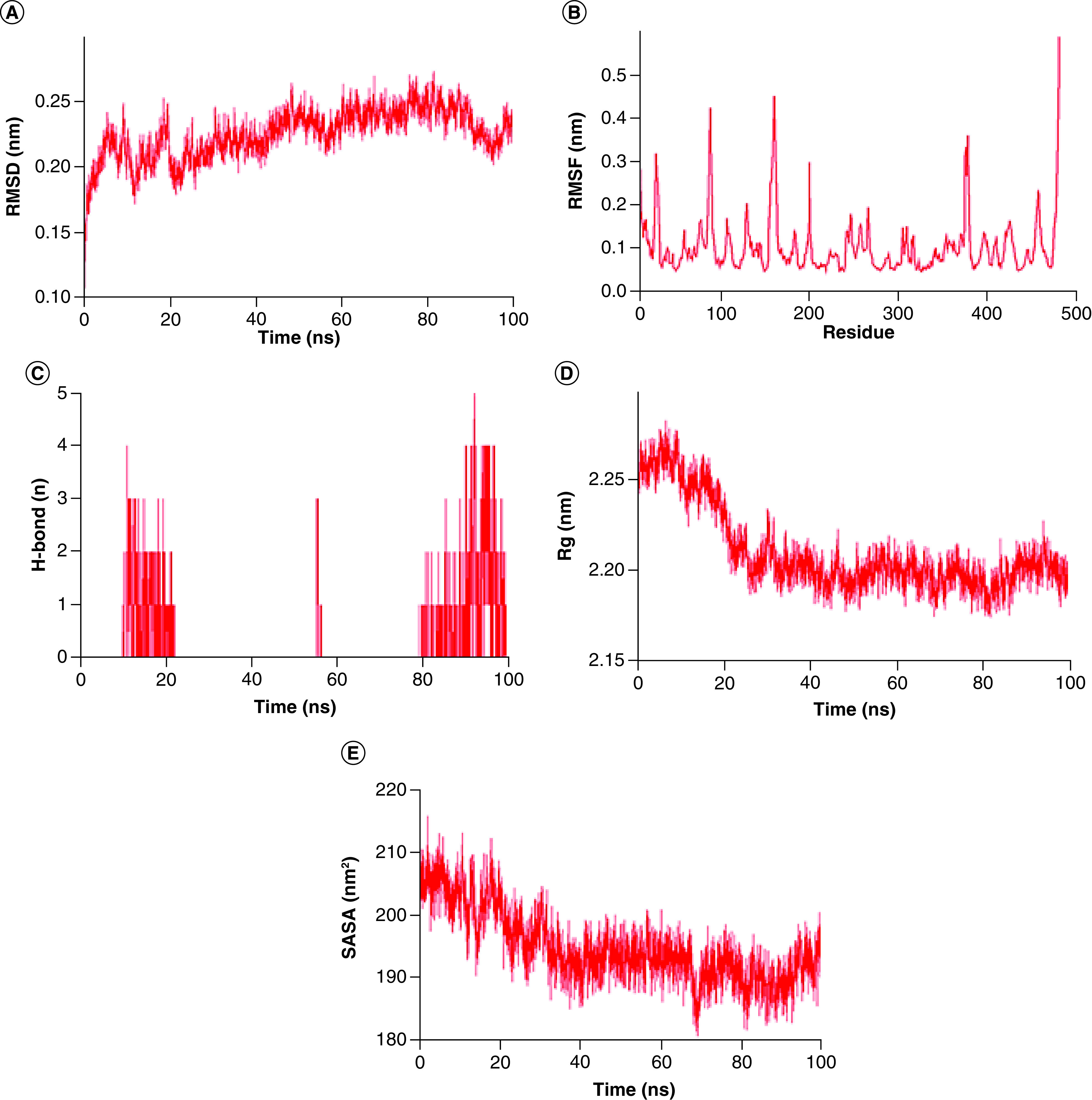
MD simulation analysis of furin–andrographolide complex protein with their molecular dynamics trajectories. **(A)** Root mean square deviation analysis of furin–andrographolide complex protein. **(B)** Root mean square fluctuation analysis of furin–andrographolide complex protein. **(C)** Total number of hydrogen bond formed between furin–andrographolide complex protein in 100 ns MD simulation. **(D)** Rg simulation analysis of furin–andrographolide complex protein. **(E)** Solvent accessible surface area of furin–andrographolide complex protein with the ordinates in Armstrong square (Å2) and the abscissa in nanosecond time (ns).

NMA is very useful in determining the confirmation of binding sites in the protein–ligand complex which can be illustrated as advanced correlating protein dynamics in their normal mode. In our study, iMODs service platform was used to perform NMA analysis on essentially selected two docked complexes including furin–andrographolide and TLR4R4-MMD2R4–andrographolide normal modes of proteins to assess their flexibility and stability performances, as shown in Figure 4 & 5, respectively. The iMODs results drove the domain's mobility toward one another due to the formation of furin–andrographolide and TLR4R4–MMD2R4–andrographolide complexes, as shown by the arrows in Figure 4A & 5A. The B-factor result values indicated that the relative amplitude of atomic shifts was in a balanced state, and they were also equivalent to RMS as assessed by NMA (Figure 4B & 5B). While deformability was calculated based on the particular deformation of a single residue, the pivots of the graph denote a high deformability zone (Figure 4C & 6C). The motion stiffness of C-alpha atoms was computed by using eigenvalues conjugated with a validated normal model. The lower the eigenvalue, the simpler is to deform the complicated structure since less energy is required. The relative eigenvalues for furin–andrographolide complex and TLR4R4–MMD2R4–andrographolide complex were determined to be 3.287932e-04 and 1.656982e-05 respectively indicating that the complexes are extremely stable (Figure 4D & 5D). Figure 4E & 5E illustrated the variance associated with each standard mode (here 20 normal modes were chosen for computation) inversely related with eigenvalue, with individual variances as displayed in red shades and cumulatively variances in green shades (shown in respective figures Figure 4E & 5E). The covariance matrix demonstrated a relationship between the pairs of residues, as indicated by blue shades, white shades and red shades which represent anticorrelated, uncorrelated and correlated pairs of residues, respectively, as shown in Figure 4F & 5F. On the other hand, an elastic network graph describes the pairing of two individual atoms bonded by springs, and each dot in the scatter graph represents a spring between the respective pair of atoms. The darker greys colour region in the graph indicates the stiffer springs, as demonstrated in Figure 4G & 5G.

### Molecular dynamic simulation of protein–ligand complexes

Conformational stability and consistency of the selected furin–andrographolide docking complex were determined using several parameters, such as RMSD, RMSF, the radius of gyration (Rg), SASA energy and hydrogen bonds through MD simulation conducted for 100 ns (Figure 6). It was observed that the furin–andrographolide complex presented a stable complex in the course of the 100 ns MD-simulation experiment. From the RMSD plot, the fluctuation was observed between 0.15 and 0.25 ranges. At the primary stage (between 0 and 35 ns), a higher degree of instability was noted in the complex while good stability was recorded after 35 to 100 ns. Fundamentally, the RMSD plot revealed the most comparative stable regions between the 60 and 90 ns simulation period (Figure 6A). Similarly, a confirmative variant of the furin–andrographolide complex ranging from 0.05 to 0.5 nm was also identified through the RMSF graph analysis. This result indicates that the compound has a close relationship at their binding pockets/interface along with a minute fluctuation over a 100 ns period (Figure 6B). Furthermore, analyses were done with five H-bond interactions present in the docking complex. Out of the five, 3–4 H-bond interactions were observed throughout the 100 ns simulation, therefore these H bonds are actually involved in the binding affinity of andrographolide and stabilizing the Furin–andrographolide complex (Figure 6C). Additionally, the Rg value measured the residual compactness and was displayed within a range between 2.15 and 2.25 nm. The minimal frequency of Rg indicated that andrographolide possesses tight binding with the target enzyme (Figure 6D). The average SASA values for the furin–andrographolide complex were observed between 180 and 210 nm^2^, indicating the absence of any significant conformational variation in the protein–ligand complex (Figure 6E). Thus, the above analyses suggest that andrographolide is efficient to form a stable complex with the important host molecules associated with SARS-CoV-2 infection and this could be utilized to control the SARS-CoV-2 infection, ironically.

### Pharmacokinetics & bioavailability of andrographolide & related phytochemicals

After confirming the efficacy of andrographolide against the protein targets, pharmacokinetic analyses were performed to check whether it is suitable for drug development or not. The possible pharmacokinetic profiles of the selected andrographolide phytochemical are displayed in Table 2. The ADMET profile indicates that the ligand possesses outstanding water solubility, Caco-2 permeability, oral bioavailability and oral intestinal absorption in the gastrointestinal (GI) tract profiles which are considered as essential factors for an ideal drug candidate. Furthermore, the ligand was found to exhibit a suitable blood–brain barrier with low molecular penetration to the central nervous system through human plasma cells. In addition, the compound was found to be non carcinogenic to the human cell with low level of AMES toxicity. The receptor binding analysis also suggests that the compound had a primary cell-binding site with estrogen, thyroid, glucocorticoid, aromatase and PPAR-γ receptors. Thus, from the pharmacokinetics and toxicity point of view, the ligand is bearing suitable drug-ability profiles.

**Table 2.** Predicted pharmacokinetics and ADMET properties of anti-SARS-CoV-2 andrographolide compound.

Andrographolide			
Property	Model name	Unit	Predicted value with absence/presence of activity
Absorption	Water solubility	Numeric (log mol/l)	-3.494
	Caco-2 permeability	Numeric (log Papp in 10^-6^ cm/s)	1.07
	Human intestinal absorption	Numeric (% absorbed)	95.357
	Skin permeability	Numeric (log Kp)	-3.794
	P-glycoprotein substrate	Categorical (yes/no)	No
	P-glycoprotein inhibitor	Categorical (yes/no)	No
Distribution	Human VDss	Numeric (log l/kg)	-0.286
	Human fraction unbound	Numeric (Fu)	0.281
	Blood–brain barrier permeability	Numeric (log BB)	-0.598
	CNS permeability	Numeric (log PS)	-2.691
	Plasma protein binding	Numeric (% absorbed)	0.536
	Subcellular localization	Numeric (% absorbed)	0.7024
	OATP1B1 inhibitior	Categorical (yes/no)	Yes
	OATP2B1 inhibitior	Categorical (yes/no)	No
	OATP1B3 inhibitior	Categorical (yes/no)	Yes
	OCT2 inhibitior	Categorical (yes/no)	No
	MATE1 inhibitior	Categorical (yes/no)	No
	BSEP inhibitior	Categorical (yes/no)	Yes
	CYP inhibitory promiscuity	Categorical (yes/no)	No
Metabolism	CYP2D6 substrate	Categorical (yes/no)	No
	CYP3A4 substrate	Categorical (yes/no)	Yes
	CYP1A2 inhibition	Categorical (yes/no)	No
	CYP2C19 inhibition	Categorical (yes/no)	No
	CYP2C9 inhibition	Categorical (yes/no)	No
	CYP2D6 inhibition	Categorical (yes/no)	No
	CYP3A4 inhibition	Categorical (yes/no)	No
Excretion	Total clearance	Numeric (log ml/min/kg)	1.183
	Renal OCT2 substrate	Categorical (yes/no)	No
Toxicity	AMES toxicity	Categorical (yes/no)	No
	Maximum tolerated dose (human)	Numeric (log mg/kg/day)	0.128
	Acute oral toxicity(kg/mol)	Numeric (log kg/mol)	2.795
	hERG I inhibitor	Categorical (yes/no)	No
	hERG II inhibitor	Categorical (yes/no)	No
	Oral rat acute toxicity (LD50)	Numeric (mol/kg)	2.162
	Oral rat chronic toxicity (LOAEL)	Numeric (log mg/kg_bw/day)	1
	Hepatotoxicity	Categorical (yes/no)	No
	Skin sensitisation	Categorical (yes/no)	No
	T.Pyriformis toxicity	Numeric (log µg/l)	0.491
	Minnow toxicity	Numeric (log mM)	1.37
	Carcinogenicity	Categorical (yes/no)	No
	Human either-a-go-go inhibition	Categorical (yes/no)	No
Receptor binding	Estrogen receptor binding	Categorical (yes/no)	Yes
	Androgen receptor binding	Categorical (yes/no)	Yes
	Thyroid receptor binding	Categorical (yes/no)	Yes
	Glucocorticoid receptor binding	Categorical (yes/no)	Yes
	Aromatase binding	Categorical (yes/no)	Yes
	PPAR gamma	Categorical (yes/no)	No

Determination of drug-likeness assets is also one of the essential parts of *in silico* drug designing. Accordingly, we have assessed the drug-likeness properties of the andrographolide from SwissADME webserver databases. The molecular mass of the andrographolide compound was 350.45 g/mol, and the Log P and Log S values were 2.45 and -3.18, respectively. We observed the existence of five H-bond acceptors and H-bond donor atoms in andrographolide with 86.99Å topological polar surface area (TPSA) and 0.55 bioavailability score derived from computational analysis (Table 3). Thus, the compound follows the ideal Lipinski rules of five parameters for an oral drug.

**Table 3.** Drug-likeness assets of andrographolide compound.

ADMET properties (Lipinki's rule of five)	
Name	Andrographolide
PubChem CID	5318517
Formula	C_20_H_30_O_5_
Molecular weight (150–500 g/mol)	350.45(g/mol)
Lipophilicity (Expressed as LogP) (≤5)	2.30
Log S (≤-10)	-3.18
H-bond donor (<5)	3
H-bond acceptor (<10)	5
Molar refractive index (40–130)	95.21
TPSA(20–120 ^0^A)	86.99 ^0^A
Class of solubility	Soluble
Violation	0
Bioavailability score	0.55

## Discussion

Nowadays, the novel coronavirus has become one of the biggest threats to humanity. This viral disease has a devastating impact on human health with an increased number of deaths, related comorbidities and socio-economic losses across the globe. Different repurposing drugs like hydroxychloroquine, chloroquine, ivermectin, doxycycline, remdesivir, oseltamivir, ritonavir, arbidol and favipiravir are being used continuously for treating this disease without their proven efficacy [43]. However, these drugs are remaining as under review and also have significant questions regarding certain serious side effects with certain medical conditions such as diabetes, heart attacks and hypertension [65]. Considering the present scenario, new therapeutics against SARS-CoV-2 is indeed the need of the hour. Especially the drug that could restrict the viral load as well as the severity raised by nonspecific inflammation of the host caused by the virus is the utmost need. To accomplish this, different natural compounds have been tested against SARS-CoV-2 virus throughout the world to get newer molecules for anti-COVID therapy. Several phytocompounds previously contributed to the treatment of other viral diseases like HIV, influenza virus and MERS-CoV virus have been emphasized for the search. Moreover, natural antiviral compounds possess limited side effects and can enhance human immunity, which is important for defeating microbial enemies [66].

We have also performed a comprehensive literature survey through PubMed and Google scholar databases to screen out natural bioactive compounds with antiviral properties from various medicinal plants [63,67]. Out of these survey reports, we found several antiviral phytochemical compounds, which were further selected based on their immunomodulatory properties against RNA viruses and used to treat related signs and symptoms associated with SARS-CoV-2 disease. Concerning this view, a natural antiviral compound, i.e., andrographolide, an active secondary metabolite of Indian ethnomedicinal plant *A. paniculata* has been selected because of its incredible medicinal properties [68]. Importantly, this compound is widely used in Ayurvedic formulation/medicine for the treatment of different human diseases [68]. This natural compound has many significant biological aspects, including anti-inflammatory, antibacterial, anti-tumor, anti-diabetic, antimalarial and hepatoprotective activity [69]. Furthermore, it has proven antiviral effects against HIV, Dengue and other viral diseases [70]. Moreover, few reports have come out showing this molecule has a direct effect on different inhibitory enzymes of SARS-CoV-2 [40]. Sa-Ngiamsuntorn *et al.* [71] conducted an experiment with andrographolide that revealed the reduction of SARS-CoV-2 burden in lung epithelial cell line Calu-3 as well as in the liver, kidney intestine and brain cell lines. In addition, Shi *et al.* [72] have shown that andrographolide directly inhibits the SARS-CoV2 Mpro. However, it has still limited data with prior *in silico* experimental results. None of the previous studies has properly evaluated the potentiality of this natural antiviral molecule against host pathogenicity as well as inflammation occurs during COVID-19 illness. In this context, the present *in silico* study depicts the potential inhibitory properties of andrographolide against the possible key host factors, i.e., ACE2, TMPRSS2, furin, Cathepsin L, IL-6 and TLR4-MD2 that are directly associated with severity of SARS-CoV-2 pathogenesis.

In SARS-CoV-2 pathogenesis, the host molecule ACE2 is the most important receptor involved in the entry of SARS CoV-2 into the host epithelial cells by serving itself as a receptor for the viral spike glycoprotein [73]. Furthermore, the ACE2 receptor protein is usually linked with cardiovascular diseases found in COVID-19 affected patients and with severe chronic diseases such as lung diseases, diabetes and heart disease [74,75]. A previous *in silico* study recently revealed that terpenoid compound andrographolide can potentially inhibit the surface receptor protein ACE2 of SARS-CoV-2 with a molecular docking score of -7.67 kcal/mol [76]. Herein, this compound reveals that it has a higher binding affinity in our experiment relative to the aforementioned *in silico* study of the human receptor ACE2 protein with a molecular docking score of -8.99 kcal/mol. Therefore, such a high binding score observed in our docking experiment suggests that andrographolide has a greater interactive effect on ACE2 receptor protein which may be an alternative way to block the SARS-CoV-2 infection. Following the interaction of host ACE2 receptor with viral S protein, priming of viral S protein is necessary for the entry of the virus particles into cells [10]. This priming of the S protein is achieved by one of the two host serine protease TMPRSS2 or Cathepsin L [77]. It has been established that priming processes are mainly mediated by TMPRSS2 enzyme but other than TMPRSS2, Cathepsin L also performs the same job. Moreover, Hoffmann and their colleagues [10] have recently demonstrated that SARS-CoV-2 can be hijacked through TMPRSS2 protease enzyme protein to prime the spike glycoprotein of coronavirus. Additionally, inhibition of TMPRSS2 by camostat or by blocking Cathepsin L could restrict the entry of SARS-CoV-2 into the cell [10]. An *in silico* study conducted by Vivek-Ananth *et al.* [9] has demonstrated that different biomolecules of Indian medicinal plants also bind with TMPRSS2 and Cathepsin L to block the cell entry of SARS-CoV-2. Herein, we have also found that andrographolide could successfully bind with TMPRSS2 and Cathepsin L with a strong molecular docking score of -8.96 and -9.50, respectively. Therefore, andrographolide can strongly restrict the priming process of S protein and also restrict the viral endocytic entry to cells. It is already known that TMPRSS2 is a kind of androgen receptor [78]. Interestingly, earlier experimental evidence revealed that andrographolide is a natural inhibitor of androgen receptors and inhibits the castration-resistant prostate cancer cells [79]. Concerning this, TMPRSS2 has received a lot of attention in the context of prostate cancer, it is expressed abundantly and increases the responses to the androgens through direct transcriptional regulation of androgen receptors (AR) [80]. Therefore, this work supports our hypothesis that inhibition of androgen receptor and androgen-regulated genes by andrographolide could block TMPRSS2 [81]. On the other hand, a recent *in vitro* analysis by Zhang *et al.* [82] revealed that, glycopeptide antibiotic teicoplanin has potential to block viral entry route of SARS-CoV-2 viruses into the cells through blocking the Cathepsin L. Besides these, Shah *et al.* [83] has also reported that oxocarbazate drug inhibits Cathepsin L protease enzyme to prevent human epithelial cells from the invasion of Ebola pseudo-type and SARS-CoV viruses. Since andrographolide has the potential to bind with Cathepsin L, it might inhibit the normal Cathepsin L activity for priming of SARS-CoV-2 S protein, and thereby inhibits the endocytosis of the virus particles in cell and infection.

The SARS-CoV-2 infection involves two indispensable cleaving processes. Apart from the above-mentioned human protease enzymes TMPRSS2 and Cathepsin L, it was noted that the cleavage site of S1or S2 subunits of SARS-CoV-2 could be recognized by the host furin enzyme for proteolysis. Furin is a kind of proprotein convertase activated by low pH and usually found near the trans-Golgi network [84]. Indeed, the proteolytic enzyme furin is essentially required for viral entry into the host cells, but it has been demonstrated that furin is well manifested in the host cells present in the respiratory tract and thus increases the viral load of SARS-CoV-2 [85]. Nevertheless, a group of scientists also highlighted that novel furin-like proteolytic enzyme could identify the sequence of spike glycoprotein at the S protein maturation site that could exert a substantial functional effect on the entry of SARS-CoV-2 [86]. Most importantly, SARS-CoV-2 S protein has randomly contained more than one furin cleavage site that might increase the infectivity of SARS-CoV-2 [15]. However, no such small-molecule inhibitor of the furin enzyme has been discovered so far. Recently, an *in* *vitro* study revealed that an anti-parasitic drug diminazene inhibits human furin protease enzyme competitively with an IC50 value of 542 ± 0.11 μM and can work against SARS-CoV-2 [15]. On this basis, the present *in silico* analysis on the antiviral compound andrographolide revealed inhibition of furin enzymatic protein with a molecular docking value of -10.54 kcal/mol (Table 1) and indicated that andrographolide can block the important physiological function of furin in the human body and could reduce the severity of SARS-CoV-2 pathogenicity. The above host proteins were also cross-docked with known selective drug ivermectin B1b. The interaction studies conducted with ivermectin B1b demonstrated similar interaction values with ACE2, Cathepsin L, and furin protein but it did not show any interaction with TMPRSS2 which we have selected as a host molecule in this study (Table 1).

Considering all the experimental prediction studies on the binding affinity of andrographolide, it was found that, the complex formed by andrographolide with furin displayed the top binding value in comparison to other docked complexes like Cathepsin L-andrographolide, TMPRRS2- andrographolide and ACE2-andrographolide (Table 1). Binding affinity is inversely related to inhibition constant (ki). Therefore, the least ki value of furin–andrographolide of 18.70 nM indicates that the complex is highly affined and that prediction was evaluated in NMA analysis. NMA study reveals that the furin–andrographolide complex has a lower eigenvalue and possesses less energy required for protein deformation leading to greater stability (Figure 4D). Besides, B factor values with higher deformability indicated higher flexibility in the andrographolide–furin complex (Figure 4B & C). Furthermore, it was also simulated for the study of molecular dynamics with 100 ns wherein RMSD and RMSF values indicated good stability in the furin–andrographolide complex (Figure 6). Therefore, an antiviral compound of interest could be used as a therapeutic choice for blocking the human proteolytic enzyme furin against SARS-CoV-2 which could emerge as a better treatment option for COVID-19. Our interpretations are also supported by a group of scientists, who proposed that andrographolide might be therapeutic against SARS-CoV-2 as it could inhibit furin proprotein convertase and block the viral fusion at host cells [87]. They proposed this idea based on their study on the inhibition of HIV infection through the blockage of furin molecule by andrographolide [88].

Although SARS-CoV-2 infection is extremely contagious, approximately 15% of total cases enter into severity [89]. This severity occurs mainly due to the induction of over inflammation. Laboratory study reveals that severity develops as pneumonia, acute respiratory distress syndrome (ARDS), acute cardiac damage, etc. [90]. It has already been revealed that all these severities develop due to a storm of inflammatory cytokines like TNF-α, IL-1β, IL-6, etc. [91]. Among these cytokines, IL-6 seems to be a crucial factor in exacerbating pulmonary disease and severity in COVID patients [92]. Overproduction of cytokines by SARS-CoV-2 enhances vascular permeability and results in invading a substantial volume of blood cells and fluids to the alveoli cells, causing serious damage to the host cells and breath inability in COVID-19 patients [93,94]. Therefore, along with antivirals, anti-inflammatory drugs are also used against SARS-CoV-2 infection. Drugs like tocilizumab [95], chloroquine [96], or azithromycin [96] are used regularly in SARS-CoV-2 infection to inhibit the cytokine storm and restrict the severity of the disease. It is known that most of the innate cytokine responses are generated through the activation of TLRs and importantly SARS-CoV-2 infection also triggers the activation of different TLR molecules to induce inflammatory responses [24,97]. Recently, it has been updated that activation of cell surface TLR4 is crucial for recognition of SARS-CoV-2 S protein [24,97]. Furthermore, mRNAs of SARS-CoV-2 are also found to be recognized by the intracellular TLRs like TLR3, TLR7, TLR8 and TLR9 to activate the downstream inflammatory impulse [98]. After activation of the membrane-bound and intracellular TLRs, NF-κB is activated and produces inflammasome which makes the cytokine storm and causes severe immunopathogenesis. In an earlier *in silico* study on NF-κB, Raghavan *et al.* [41] showed an effective binding activity of andrographolide with NF-κB protein [41]. Our result also shows a similar pattern with nearby binding values of andrographolide binding to NF-κB protein (Supplementary Table 1). Furthermore, many scientific reports have demonstrated that andrographolide has also important pharmacological activities like an anti-inflammatory response to attenuate the innate immune response in various diseases [99,100]. On that basis, our *in silico* study with andrographolide reveals that this phytocompound possesses strong binding affinity against IL-6 as well as TLR4-MD2 protein. Taking clue from the hypothesis, we have further checked the effect of ivermectin against the mentioned inflammatory proteins. However, ivermectin only interacts with the TLR4–MD2 complex but not with IL-6 regulatory protein. Thus, andrographolide appears to be the potential immunomodulatory agent that is expected to inhibit both pro-inflammatory cytokine IL-6 as well as TLR4-MD2 proteins. In support, different experimental data have shown that andrographolide inhibits TLR4–Myd88–NFκB pathway and inhibits inflammasome activation as well as innate proinflammatory cytokine production [97,101–103]. Furthermore, it was also experimentally determined that andrographolide inhibits IL-6 expression in prostate cancer cells and limits the growth of prostate cancer [104]. Therefore, andrographolide might be a useful molecule for attenuation of innate pro-inflammatory cytokine storm as well as inhibition of the most important pathological cytokine IL-6 in SARS-CoV-2 infected individuals. Subject to the above study, the most effective way of andrographolide in diminishing immunopathogenic consequences was achieved through binding affinity prediction and shown that TLR4-MD2 is a promising way for TLR4–andrographolide complex as displayed lowest ki value in comparison to IL-6-andrographolide complex. In support, NMA, as well as MDS studies, revealed that complex has greater stability and flexibility (Figure 5), which in conclusion recommends andrographolide possesses therapeutic potentiality in blocking TLR4-MD2-mediated inflammatory pathophysiological consequences of COVID-19 patients. Altogether, it notifies that andrographolide targets more relevant interaction among host-associated target proteins that are responsible for SARS-CoV-2 infection, as well as pathogenesis, indicating it has an independent association with SARS-CoV-2 and works as host-directed therapy.

**Figure 7.**
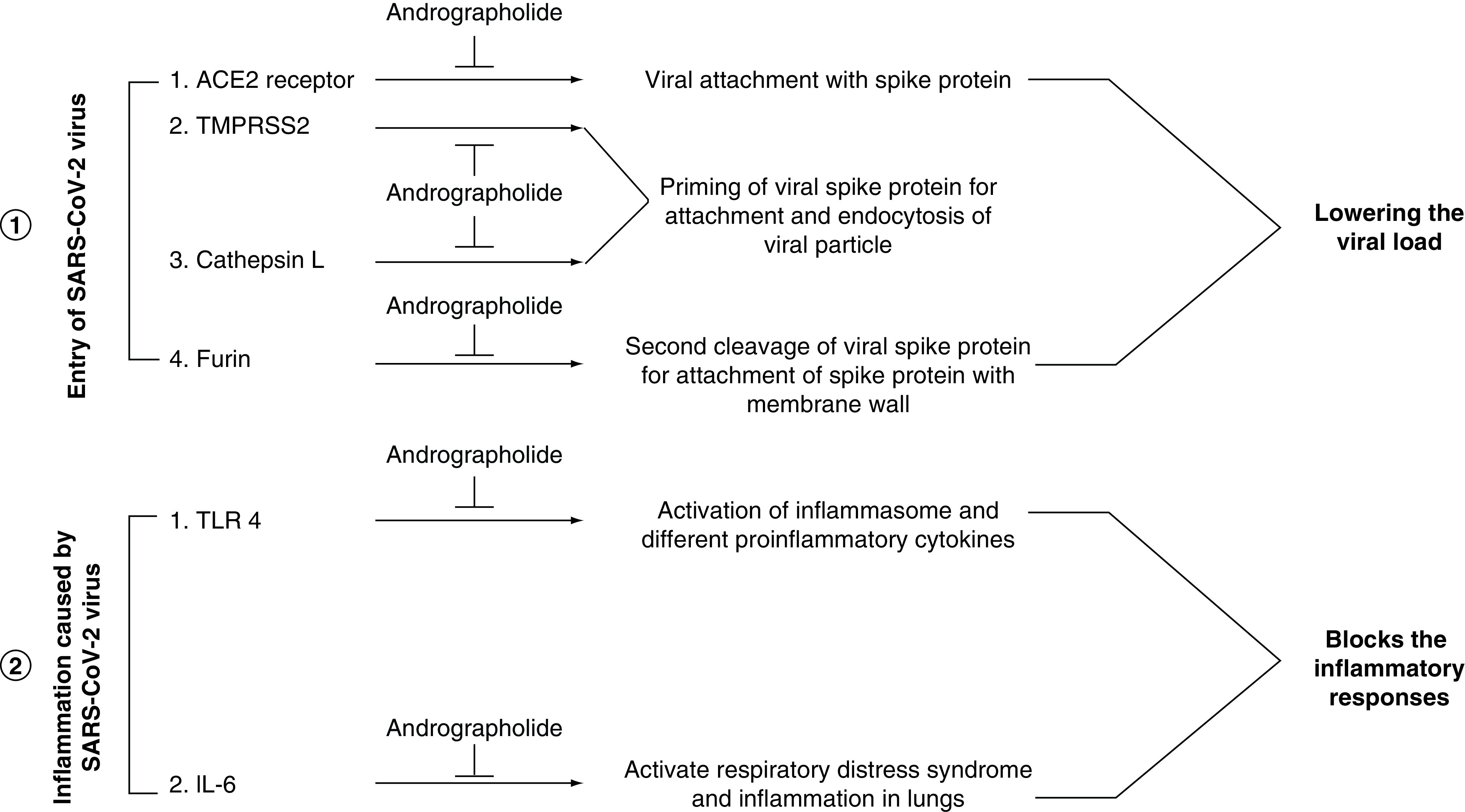
Scheme depicting possible mechanism of anti-SARS-CoV-2 action of andrographolide.

In the twenty-first century, pharmacokinetic analysis and ADMET assessment of phytochemical molecules have sparked the attention of researchers because of highly relevant strategies for reducing the high expenditures on drug production processes. Besides these, it also helps to minimize the main drug-related risk factors during the drug development process and provides useful knowledge regarding the suitability of a molecule to become a therapeutic or not [105]. A recent *in vitro* investigation by Sa-Ngiamsuntorn *et al.* [71] revealed that andrographolide was non cytotoxic to different human cell lines, including liver (HepG2 and imHC), brain (SH-SY5Y), kidney (HK-2), intestine (Caco-2) and lung (Calu-3) with CC_50_ values of 81.52, 44.55, 13.19, 34.11, 52.30, 58.03 μM, respectively. In another report Palit *et al.* [87], have demonstrated that andrographolide does not cause any harmful alteration in AMES toxicity at a tolerable dose of 0.128 log mg^-1^ kg^-1^ day^-1^ meant for human consumption [88]. The LD50 value of andrographolide is 2.162 mol kg^-1^. At this dose, the phytocompound does not inhibit any hERG-I or hERG-II enzymes and does not induce any hepatotoxicity or skin irritation [87]. On the other hand, our *in silico* toxicity prediction was found to be compatible with the above evidence and demonstrates that the molecule has a drug bioavailability score as shown in Table 2. Although the potential of this molecule has been explored but still its solubility and bioavailability differ significantly. Using spray-drying, nanoemulsion, microsphere, microemulsion, liposome, niosome, and nanoformulation of the molecule increases the half-life of andrographolide [106–108]. Furthermore, Yen, with his colleagues, experimented and found that silver-loaded andrographolide nanoparticles enhance bioavailability by 594.3% and are efficient against pro-inflammatory cytokines (eg, IL-1β, IL-6 and TNF-α) and the chemokine IL-8 [106]. Similarly, in an *in vitro* breast cancer model, encapsulating andrographolide in PLGA nanoparticles helps to overcome solubility difficulties and allowed for continuous drug release [107]. The use of hydroxypropyl-β-cyclodextrin (HP-β-CD) formulated andrographolide molecule increased its bioavailability by 1.19 percent [109]. With previewing of the above reports, the molecule also displays a lower molecular weight with low molar refractive index, insignificant lipophilicity effects in the body and exhibits excellent TPSA (topological polar surface area) with a higher absorption profile. Furthermore, this compound does not interfere with any beta-blockers, anti-depressants, anti-arrhythmic or anti-hypersensitive opioids by activation of the CYP2D6 enzyme. Moreover, it does not interfere with the metabolism of anti-seizure, anti-clotting, type II diabetes, anti-inflammatory or anti-hypertensive compounds that are regulated by the CYP2D9 enzyme identified from predicted AMES toxicity analysis. In addition, it was also noted that it does not inhibit any fatty acids, steroid oxidations or xenobiotics for hormone synthesis through inhibition of CYP3A4 enzyme. As a result of this toxicity prediction, andrographolide qualifies with zero infringement of all Lipinski's rule of five (Table 3), and is considered as bio-safe with a potential therapeutic molecule against SARS-CoV-2 disease. The results of *in silico* investigation employing andrographolide with host proteins interactions are shown in a schematic diagram to depict each phase of the process (Figure 7).

## Conclusion

Our experimental data acquired from *in silico* studies may provide new insight regarding the candidature of the natural compound andrographolide for the therapeutic intervention of SARS-CoV-2 through host-directed therapy. Most importantly, in this growing condition of mutations in SARS-CoV-2, host-directed therapy is a novel way to approach treatment. In this direction, the most unique part of our work is that it specifies a single molecule, i.e., andrographolide which interacts with all the major host proteins associated with viral infection as well as involved in inflammation. Thereby, data with andrographolide are evident *in silico* experiments, single-molecule andrographolide has a different magnitude of interaction with many host receptors responsible for SARS-CoV-2 infection and pathogenicity. Additionally, many other works showed that andrographolide has a direct blocking effect on viral proteins. Taken together it might be said that this molecule could resist the SARS-CoV-2 infection prominently and also restrict the severity of the disease caused by over inflammation. Therefore, andrographolide can be used as a future drug molecule for SARS-CoV-2 infection, but it requires vivid *in vitro* and clinical studies to validate this compound completely against the SARS-CoV-2 virus.

**Summary points**

- This scientific study provides therapeutic efficacy of andrographolide of *Andrographis paniculata* as potential drug candidate for suppressing SARS-CoV-2 infection via interrupting the connection between host and virus.
- Docking was performed in between andrographolide and different human–host protein recipients associated with inflammatory pathogenesis of SARS-CoV-2 like furin, TMPRSS-2, ACE-2, Cathepsin L, TLR4-MD2 and IL-6 cytokine.
- The molecular interactions between andrographolide with pro-enzyme furin protein were analyzed through normal mode analysis and molecular dynamic simulation.
- Binding efficacy of andrographolide against the functionally important host receptors and viral proteins of SARS-CoV-2 pathogenesis clearly indicate the potentiality of this compound to block all the entry point of SARS-CoV-2.
- Notably, this drug effectively binds to inflammatory regulators, such as TLR4-MD2 and IL-6 cytokine which could be useful to mitigate SARS-CoV-2 induced ‘cytokine storm’ pathogenicity.
- Pharmacokinetic and pharmacodynamics properties of andrographolide were found to be comparatively non-toxic and appears to be safe as a efficient herbal drug in COVID-19 remedy.
