# Supplementary material for: Andrographolide induces anti-SARS-CoV-2 response through host-directed mechanism: an in silico study
Source: Future Virol. 2022 Jul 4:10.2217/fvl-2021-0171. doi: 10.2217/fvl-2021-0171 (PMC9254363; doi:10.2217/fvl-2021-0171)
Supplement: Supplementary file 1 [file supplementary_data.docx]

**Supplementary Table 1.** Comparative docking analysis of andrographolide against NF-κB and viral main protease protein involved in SARS-COV-2 pathogenesis.

| Scientific name | Natural product found in India  West Bengal /Odisha name | Compound Name | Receptor | Types of Bonds | No. of Bonds formed | Amino acid residues | Bond Length (A ֩) | Free energy of binding affinity (ΔG) (kcal/mol) | Inhibition constant (Ki) in nM (nanomolar)/pM (picomolar) |
| --- | --- | --- | --- | --- | --- | --- | --- | --- | --- |
| *Andrographis paniculata* (Burm.f.) Nees | Kalmegh/ Bhunimba | Andrographolide | Main protease | Hydrogen | 5 | THR111 | 1.8599 | -10.42 | 23.01 nM (nanomolar) |
|  |  |  |  |  |  | GLN110 | 1.92188 |  |  |
|  |  |  |  |  |  | GLN110 | 2.04435 |  |  |
|  |  |  |  |  |  | GLN110 | 1.99979 |  |  |
|  |  |  |  |  |  | ARG105 | 3.13288 |  |  |
|  |  |  | I-kappa-B-alpha NF-kappa-B | Hydrogen | 8 | GLN29 | 2.66479 | -12.41 | 804.01 pM (picomolar) |
|  |  |  |  |  |  | ILE224 | 2.08925 |  |  |
|  |  |  |  |  |  | GLN241 | 1.89841 |  |  |
|  |  |  |  |  |  | ILE224 | 2.04917 |  |  |
|  |  |  |  |  |  | ILE224 | 2.02844 |  |  |
|  |  |  |  |  |  | GLU222 | 2.20363 |  |  |
|  |  |  |  |  |  | GLU225 | 3.1368 |  |  |
|  |  |  |  |  |  | GLU49 | 3.08678 |  |  |
|  |  |  |  |  |  | SER108 | 2.60 |  |  |
|  |  |  |  |  |  | LEU39 | 1.86 |  |  |
|  |  |  |  |  |  | GLN102 | 3.20 |  |  |
